# An unexpected but interesting response to a novel therapy for malignant extragastrointestinal stromal tumor of the mesoileum: a case report and review of the literature

**DOI:** 10.1186/1477-7819-11-174

**Published:** 2013-08-05

**Authors:** Hengping Li, Jun Li, Xingwen Li, Yaqiong Kang, Qiang Wei

**Affiliations:** 1 Department of Urology, West China Hospital, Sichuan University, Chengdu, Sichuan, People’s Republic of China; 2 Department of Surgical Oncology, Gansu province cancer hospital, Lanzhou, Gansu, People’s Republic of China; 3 Pathological Department, Gansu province cancer hospital, Lanzhou, Gansu, People’s Republic of China

**Keywords:** Extragastrointestinal stromal tumor, Chemotherapy, Neoadjuvant chemotherapy, Imatinib

## Abstract

**Background:**

Gastrointestinal stromal tumors (GISTs) are the most common mesenchymal tumors of the gastrointestinal tract. Extragastrointestinal stromal tumors (eGISTs) of the mesoileum are extremely rare and are usually treated with surgery combined with imatinib therapy.

**Case presentation:**

We present the case of a 43-year-old man who developed a large eGIST in the mesoileum. Abdominal/pelvic computed tomography revealed a large heterogeneous mass with cystic and solid components that measured 20.0 × 12.0 × 8.0 cm. Three cycles of neoadjuvant chemotherapy with epirubicin, cyclophosphamide and hydroxycamptothecin; en bloc resection; and three more cycles of adjuvant chemotherapy with the same regimen and drugs resulted in five years of disease-free survival without any symptoms.

**Conclusions:**

Although imatinib treatment is usually chosen for eGISTs, resistance to imatinib remains a concern; these patients may receive neoadjuvant or adjuvant chemotherapy. In case of the former, further treatment, that is, surgery or adjuvant chemotherapy, depends on tumor response to the neoadjuvant chemotherapy. In addition, this treatment for eGIST is not only beneficial but also economical for patients compared with imatinib. A novel treatment approach that combined neoadjuvant chemotherapy, surgery and adjuvant chemotherapy resulted in long-term survival in our patient, thus showing promise as a potential therapy for eGISTs.

## Background

Although gastrointestinal stromal tumors (GISTs) are rare, they are the most common mesenchymal tumor of the gastrointestinal (GI) tract. However, there are a few clinical reports of extragastrointestinal stromal tumors (eGISTs) sharing the morphological, immunological, molecular and genetic traits of GISTs and originating in areas external to the GI tube [1,2], such as the mesentery, omentum, vagina, inguinal hernia sac, rectovaginal septum, ovary, pleura, scrotum, seminal vesicles and abdominal wall [3-10]. To the best of our knowledge, cases of eGISTs originating in the mesoileum have been rarely reported. Initial reports have shown the ineffectiveness of conventional chemotherapy and radiotherapy for GISTs (<10% response) [1], although some reports have shown moderate effectiveness of en bloc resection with adjuvant imatinib therapy for eGISTs [11-13]. However, further evidence from a larger number of cases is required to clarify the effectiveness of this treatment regime. Other reports have demonstrated the development of resistance to imatinib in patients with metastases or recurrent GISTs that were then left untreated, thus increasing the risk of death [14,15]. Here we present a case of eGIST that showed an unexpected but interesting response to a novel treatment approach that combined neoadjuvant chemotherapy, surgery and adjuvant chemotherapy in a 43-year-old man.

## Case presentation

A 43-year-old man was referred to our institute after computed tomography (CT) performed for lower abdominal pain and postprandial fullness at a local hospital revealed a heterogeneous mass comprising mixed cystic and solid components and measuring 20.0 × 12.0 × 8.0 cm. Lymph node involvement or metastasis was not detected. Abdominal ultrasound confirmed the findings by revealing a heterogeneous mass in the same location. The patient gave no history of bowel habit changes, fever, chills, nausea, trauma, past surgeries or family history of malignancy.

Physical examination revealed slight abdominal distension and tenderness. Palpation revealed a large, nonpulsatile, fixed, moderately tender abdominal mass. The results of tests for examining blood, stool and serum tumor markers (CA-19-9, CA-125 and CEA) were unremarkable, as were chest X-ray and rectal examination findings.

**Figure 1:**
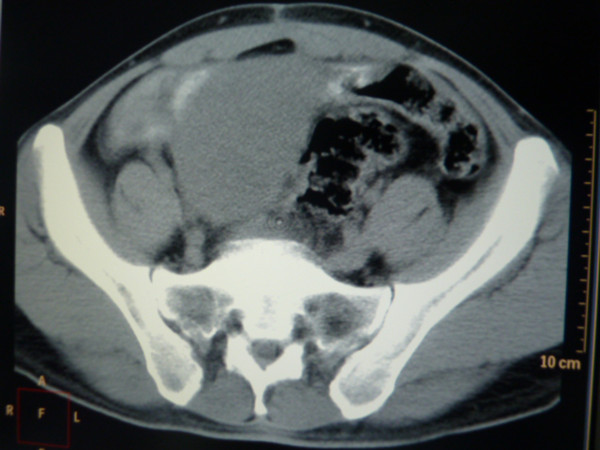
**CT Imaging after three cycles of neoadjuvant chemotherapy.** CT, computed tomography.

**Figure 2:**
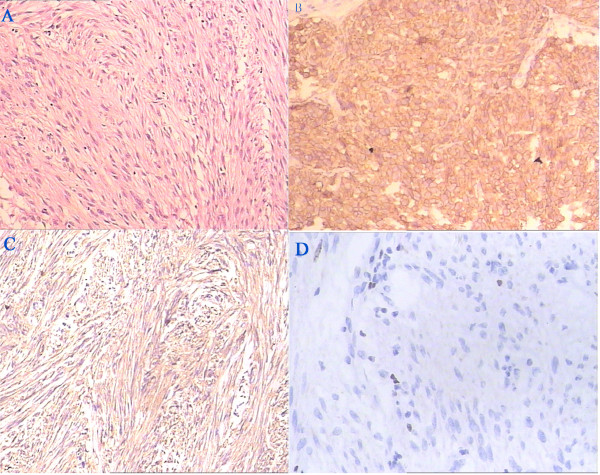
**Immunohistochemical feature of tumor.** A standard H & E assay of tumor cells is observed in **(A)**, tumor cells characteristically express CD117 **(B)**, CD34 **(C)**, and ki67 **(D)**.

**Figure 3:**
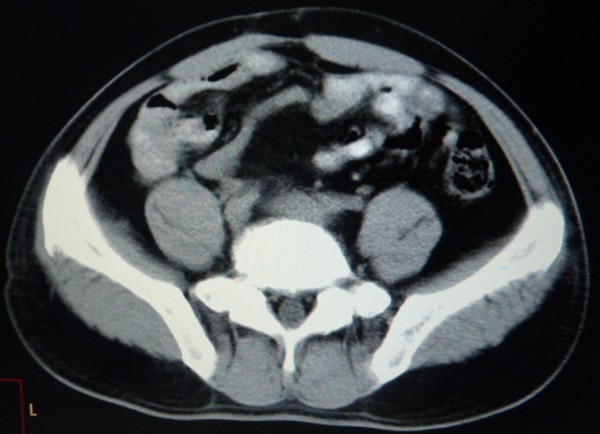
**CT Imaging in follow-up within fifth year.** CT, computed tomography.

Laparotomy revealed a large, brownish mass adherent to the colon, bladder and abdominal wall. These findings in addition to difficult hemostasis prevented resection of the mass. A biopsy sample obtained during the laparotomy procedure revealed inflammatory granulomas and necrosis. Fine needle aspiration cytology suggested the tumor to be a sarcoma with indeterminate subclassification. On the basis of the above findings, the patient was administered three cycles (every 28 days) of intravenous neoadjuvant chemotherapy with 40 mg/m2 epirubicin, 500 mg/m2 cyclophosphamide and 15 mg hydroxycamptothecin in a day. Subsequent CT revealed the mass to have diminished in size, now measuring 6.5 × 5.0 × 5.0 cm (Figure 1). This enabled en bloc resection of the mass. The gross specimen was a lobulated, grayish-white soft tissue mass measuring 6.5 × 5.0 × 4.5 cm. Histopathological examination revealed atypical spindle cells in a whorled and paliform array. The average mitotic count was 3 mitoses per 50 high power fields (HPFs). Immunohistochemical staining was diffusely and strongly positive for CD117, CD34 and Ki-67 (>10%) (Figure 2); weakly positive for S-100; and negative for desmin and smooth muscle actin (SMA). Three cycles of chemotherapy identical to those administered as neoadjuvant chemotherapy were administered after surgery. The patient exhibited five-year disease-free survival without any symptoms (Figure 3).

## Discussion

GISTs originate from the interstitial cells of Cajal or their stem cell precursors and account for approximately 5% of GI tract malignancies [1]. However, a few reports have documented eGISTs with properties similar to those of GISTs and arising from multiple extragastrointestinal sites [1-10]. Despite the presence of two controversial hypotheses concerning the origin of eGISTs, the diagnostic tests, immunohistochemical staining and large tumor size suggested a primary malignant eGIST of the mesoileum, an extremely rare entity. A majority of GISTs are comprised of a uniform cell type, either spindle-shaped, epitheloid or mixed cell type [16]. Positive immunohistochemical staining for CD117 is a defining feature of GISTs [17] and CD34 has been proposed as a reproducible marker [1,14]. Some reports, however, showed platelet-derived growth factor receptor-α (PDGFRA) gene mutations in eGISTs negative for CD117, also showed a strong correlation between an increased Ki-67 labeling index and a poor prognosis [18,19]. In our patient, histopathological examination revealed a tumor comprising atypical spindle cells in a whorled paliform array. Immunohistochemical staining revealed diffuse and strong positivity for CD117, CD34 and Ki-67. According to the findings at the first surgery, as well as pathological findings, the patient was at a high risk of recurrence despite the lower rate of karyokinesis.

The current therapy for GISTs and eGISTs is en bloc resection with adjuvant imatinib mesylate therapy. Extensive lymphadenectomy is not recommended because of the low incidence (<10%) of lymph node metastases. However, 50% of patients develop recurrent disease within two years, even after en bloc resection [20]. Increased cellularity, mitotic rate (>5/50 HPFs) and Ki-67 labeling index (>10%), necrosis, cyst formation and tumor size are parameters for evaluating GISTs, of which the first three are predictors of poor prognosis [21,22]. A mitotic rate of >2/50 HPFs, increased cellularity, and necrosis, and cyst formation have also been found to indicate aggressive behavior [23]. In our case, an increased mitotic rate (>3/50 HPF) and Ki-67 labeling index (>10%), necrosis, cyst formation and tumor size (>5 cm) all conferred a higher risk for recurrence and a worse prognosis. Initial reports indicated that conventional chemotherapy and radiotherapy were ineffective against GISTs (<10% response) [1]. Some reports showed success in cases in which the tumor was managed with en bloc resection combined with adjuvant imatinib therapy. However, resistance to imatinib has been reported in patients with metastases or recurrences [11-13]. A few reports have demonstrated the efficacy of sunitinib in patients resistant to imatinib [11-13], but further studies are needed for confirmation. No method has been very effective in patients resistant to imatinib [14,15]. Therapy for eGISTs is similar to that of GISTs. Our patient was initially diagnosed with sarcoma and was administered three cycles of neoadjuvant chemotherapy used for sarcoma, resulting in a positive chemical response. Following en bloc resection, three more cycles of the same chemotherapy were administered (considering first therapeutic efficacy and economic benefits). Under close follow-up, the patient remains free of disease at five years. The possible mechanisms could be considered as follows: 1) antibiotics administered after the first surgery relieved inflammation and decreased tumor volume, probably increased the patient’s appetite and immunological capability of resistance to the tumor according to the first postoperative results; some reports also showed that the antibiotics indirectly exert their beneficial effects by immunomodulation [24]; and 2) the cytotoxic chemotherapy effect on different cell cycles inhibited the formation of tumorous DNA and RNA and killed tumor cells. Yet, the molecular mechanisms responsible for the effects of eGISTs cell functions remain unclear.

## Conclusions

There are too few data on eGISTs to make a final conclusion regarding treatment, prognosis and recurrence. Although imatinib treatment is usually chosen for eGISTs, resistance to imatinib remains a concern; these patients may receive neoadjuvant or adjuvant chemotherapy. In the case of the former, further treatment (that is, surgery or other treatments) depends on tumor response to the initial chemotherapy. In addition, this treatment for eGIST is not only beneficial but also economical for patients compared with imatinib. A novel treatment approach that combined neoadjuvant chemotherapy, surgery and adjuvant chemotherapy resulted in long-term survival in our patient, thus showing promise as a potential therapy for eGISTs.

## Consent

Written informed consent was obtained from the patient for publication of this case report and any accompanying image.

## Abbreviations

CT: Computed tomography; eGIST: Extragastrointestinal stromal tumor; GIST: Gastrointestinal stromal tumor; H & E: Hematoxylin and eosin; HPF: High power field; SMA: Smooth muscle actin.

## Competing interests

The authors declare that they have no competing interests.

## Authors’ contributions

QW, JL, XL and HL participated in the care of the patient. HL performed the literature review and drafted the manuscript. YK obtained the pathological data. QW assisted in revising the manuscript. All authors read and approved the final manuscript.
